# Structural Variations in Articular Cartilage Matrix Are Associated with Early-Onset Osteoarthritis in the Spondyloepiphyseal Dysplasia Congenita (Sedc) Mouse

**DOI:** 10.3390/ijms140816515

**Published:** 2013-08-09

**Authors:** David W. Macdonald, Ryan S. Squires, Shaela A. Avery, Jason Adams, Melissa Baker, Christopher R. Cunningham, Nicholas B. Heimann, David L. Kooyman, Robert E. Seegmiller

**Affiliations:** 1 Department of Physiology and Developmental Biology, Brigham Young University, Provo, UT 84602, USA; E-Mails: davidmacdonald87@gmail.com (D.W.M.); rsquires86@gmail.com (R.S.S.); muy.savery@gmail.com (S.A.A.); jandhadams1@juno.com (J.A.); melssia27@yahoo.com (M.B.); cunningham22554@gmail.com (C.R.C.); nbrook7@aol.com (N.B.H.); robert_seegmiller@byu.edu (R.E.S.); 2 College of Dental Medicine, Roseman University of Health Sciences, South Jordan, UT 84095, USA

**Keywords:** spondyloepiphyseal dysplasia congenita (*sedc*), proteoglycan, animal model of osteoarthritis, Col2a1, type II collagen, articular cartilage, extracellular matrix

## Abstract

Heterozgyous spondyloepiphyseal dysplasia congenita (*sedc*/+) mice expressing a missense mutation in col2a1 exhibit a normal skeletal morphology but early-onset osteoarthritis (OA). We have recently examined knee articular cartilage obtained from homozygous (*sedc/sedc*) mice, which express a Stickler-like phenotype including dwarfism. We examined *sedc/sedc* mice at various levels to better understand the mechanistic process resulting in OA. Mutant *sedc/sedc*, and control (+/+) cartilages were compared at two, six and nine months of age. Tissues were fixed, decalcified, processed to paraffin sections, and stained with hematoxylin/eosin and safranin O/fast green. Samples were analyzed under the light microscope and the modified Mankin and OARSI scoring system was used to quantify the OA-like changes. Knees were stained with 1C10 antibody to detect the presence and distribution of type II collagen. Electron microscopy was used to study chondrocyte morphology and collagen fibril diameter. Compared with controls, mutant articular cartilage displayed decreased fibril diameter concomitant with increases in size of the pericellular space, Mankin and OARSI scores, cartilage thickness, chondrocyte clustering, proteoglycan staining and horizontal fissuring. In conclusion, homozygous *sedc* mice are subject to early-onset knee OA. We conclude that collagen in the mutant’s articular cartilage (both heterozygote and homozygote) fails to provide the normal meshwork required for matrix integrity and overall cartilage stability.

## 1. Introduction

Comprised of hyaline cartilage and found in diarthrodial joints, articular cartilage is unparalleled in the body in its tensile and viscoelastic properties. These two distinctive features are attributed primarily to the presence of proteoglycan and collagen macromolecules in the extracellular matrix (ECM) [1–5]. While surprisingly resilient, articular cartilage’s sole cell type—the chondrocyte—occupies merely 5% of total cartilage volume, indicating an essential interdependence with the ECM [6]. In order to maximize cell-matrix interaction, chondrocytes are spatially separated, non-motile, and organized into columnar arrays with cell density decreasing from superficial to deep [7–10]. This positioning is instrumental in the proper metabolism and secretion of extracellular and pericellular matrix components [10].

Composed of synthesized homotrimer propeptide α1(II) subunits, type II collagen is the predominant collagen found in articular cartilage [11,12]. In the endoplasmic reticulum and Golgi of the chondrocyte, the proα1(II) chains associate into triple helixes through electrostatic interactions and inter-chain disulfide bonding [11–13]. After folding and modification, the pro-fibrils are secreted into the ECM where they are integrated and interconnected with other type II collagens forming an abundant collagen meshwork [12,14–18]. The importance of collagen in the ECM for proper structure and functioning of articular cartilage is evident in that it comprises 50% of the dry weight of this important connective tissue [19–21].

Also originating in the chondrocyte, aggrecan proteoglycans are composed of a core protein covalently surrounded by sulfated glycosaminoglycans (GAG) [22]. Following synthesis the proteoglycans are secreted from the cell to the ECM where they interact with the structured collagen framework [23]. The anionic charges of the glycosaminoglycans interact with cationic sodium. This increase in sodium concentration in turn attracts water, thereby hydrating and stabilizing the articular cartilage and providing its compressive features [24–26].

Despite the effectiveness of articular cartilage in resisting compressive forces and torsion, it is easily damaged and difficult to repair, primarily due to its avascular nature [2,27,28]. Damage can be induced by external physical factors such as traumatic injury. Articular cartilage can also be destructively altered by genetic mutations that affect the macromolecular components, which compromises the integrity of the articular cartilage [1]. It has become increasingly clear that any compromise to the integrity of the ECM by altering either of the above two macromolecular components results in osteoarthritis-like consequences and loss of function [1].

Spondyloepiphiseal dysplasia congenita (*sedc*) is one such mutation found in both mouse and humans, which alters articular cartilage and long bone development [29,30]. *Sedc* is a missense mutation in the Col2a1 gene that results in additional disulfide bridges forming in type II collagen. This addition greatly affects the necessary association and formation of the α1 chains and the resulting triple helix [29,30]. As reported by Fernandes and Eyre, pepsin experiments in *sedc* mice showed disulfide bonding in type II collagen, whereas wild type mice lacked such bonds [30].

Our previous study of the *sedc* mutation was limited to the heterozygous (*sedc*/+) OA model in which case the skeletal phenotype is normal—presumably due to the protective effect of the wild type gene product within the growth plate. In the present study we employed the homozygous (*sedc/sedc*) OA model, which expresses dwarfism in addition to the OA-like effect on articular cartilage. We anticipated that the homozygote would provide useful information regarding the relative importance of ECM integrity in understanding the predisposition of articular cartilage to OA-like degradation.

Management strategies of articular cartilage injuries or defects in children and young adults are controversial and problematic [31,32]. Increased understanding of the ECM defect will enhance our understanding of OA and its therapeutic intervention in the human juvenile SEDC phenotypes such as Stickler syndrome [10,33,34], Kniest dysplasia [34], spondyloperipheral dysplasia [35], achondrogenesis II-hypochondrogenesis [36], and spondyloepiphyseal dysplasia [37]. Indeed, these human models shed light on the relationship between the ECM and OA. In the present study we have demonstrated unequivocally the presence of structural alterations in the ECM that are almost certainly involved in the processes resulting in OA.

## 2. Results and Discussion

### 2.1. The *sedc/sedc* Mouse Shows Unique Histological Variations in Articular Cartilage

#### Wildtype control (+/+)

**Figure 1:**
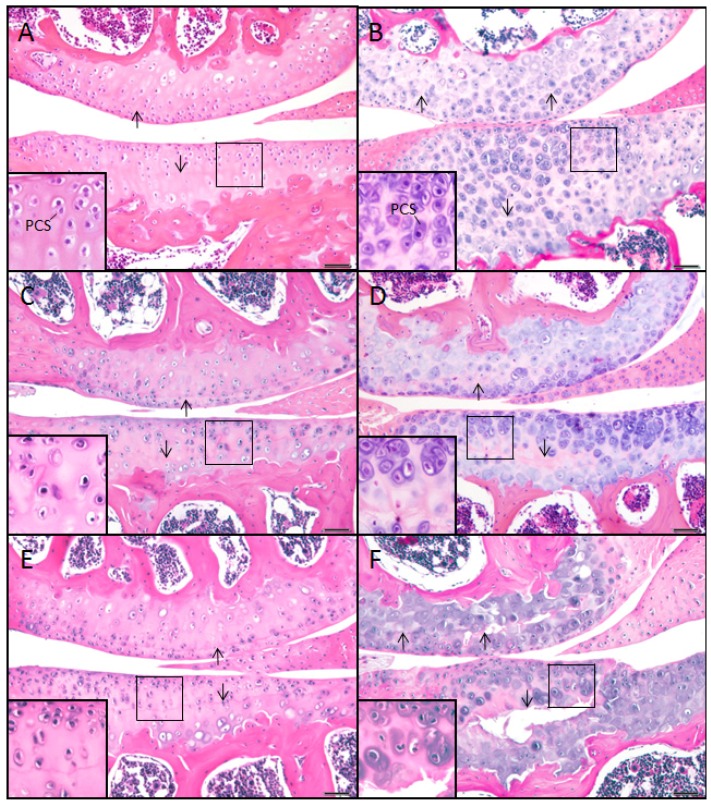
H & E staining of knee articular cartilage. Osteoarthritic degradation of cartilage is shown in *sedc/sedc* (**B**,**D**,**F**) compared with +/+ murine knee joints (**A**,**C**,**E**) at 2 (**A**,**B**), 6 (**C**,**D**), and 9 (**E**,**F**) months of age. As early as 2 months the homozygote displays an increase in cartilage thickness (superficial to deep) and chondrocytes surrounded by an enlarged basophilic pericellular space (PCS). We noted a normal smooth non-fibrillated superficial region in the mutant at all ages, just as in the case of the control. However, in the mutant, degradation was most notable within the region of the tidemark (arrows). We note that the tidemark is observed as a less distinct acellular region in the mutant relative to the WT. It also appears to be a region in which fissuring (horizontal separation) occurs. Large pane H & E-stained images were taken at 200× magnification; insets at 400× (Bar = 50 μm).

Articular cartilage of the wild-type mouse at 2, 6, and 9 months of age showed ample ECM, which stained pink or acidophilic with eosin, and subchondral bone, which stained darker pink (Figure 1A,C,E). The chondron, *i.e.*, chondrocyte and its surrounding pericellular space (PCS), was identified by the lacunar space usually containing a chondrocyte. The nucleus stained purple (basophilic) with hematoxylin as well as the immediately surrounding PCS, which often showed a thin basophilic rim presumed to be proteoglycan (Figure 1A, inset). The location of the tidemark on the condyle of the femur was close in proximity to the articular surface, while the tidemark on the tibial plateau was typically located midway between the articular surface and the subchondral bone (arrows, Figure 1A,C,E).

#### Mutant (sedc/sedc)

**Figure 2:**
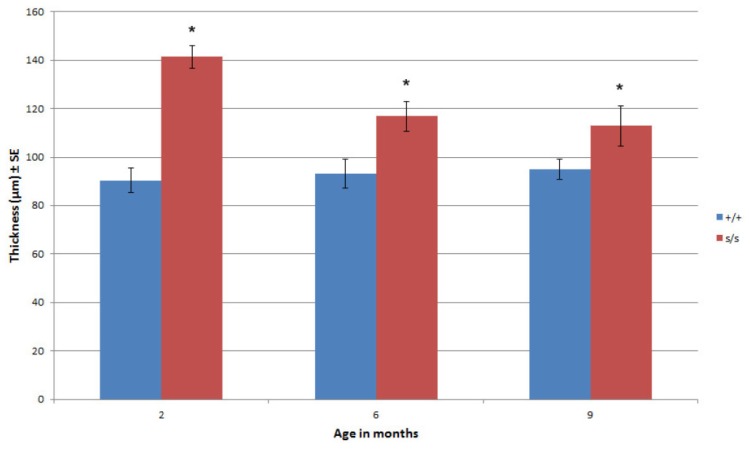
Histological measurements at 200× magnification confirmed the increased articular cartilage thickness in *sedc/sedc* compared with +/+ mice at 2, 6 and 9 months of age (*p < 0.05).

In comparison with age-matched controls at 2, 6 and 9 months, there were noticeable differences in the articular cartilage of the mutant mouse. Overall, the ECM exhibited more basophilic staining when compared with control, which was due to an increase in basophilia within the PCS (Figure 1B*vs.* 1A, inset). The location of the tidemarks on the femoral condyle and tibial plateau while less distinct in the mutant were similar to that of control at all ages. Area measurements of articular cartilage at 2 and 6 months (superficial to deep) demonstrated that the mutant has significantly more cartilaginous tissue compared with the control due to the universal expansion of the PCS and not to an increase in cell number. This expansion was seen in the vertical dimension (Figure 1B*vs.* 1A, inset). At 6 months the tidemark in the mutant was noted as an area of decreased cellularity. At 9 months this region showed horizontal fissuring often extending vertically to the superficial region. Histological measurement confirmed a significant difference in articular cartilage thickness in *sedc/sedc* compared to +/+ mice (Figure 2).

### 2.2. The *sedc/sedc* Mouse Shows Increased Safranin O Staining in the PCS Confirming Presence of Basophilic Proteoglycan

#### Wildtype control (+/+)

**Figure 3:**
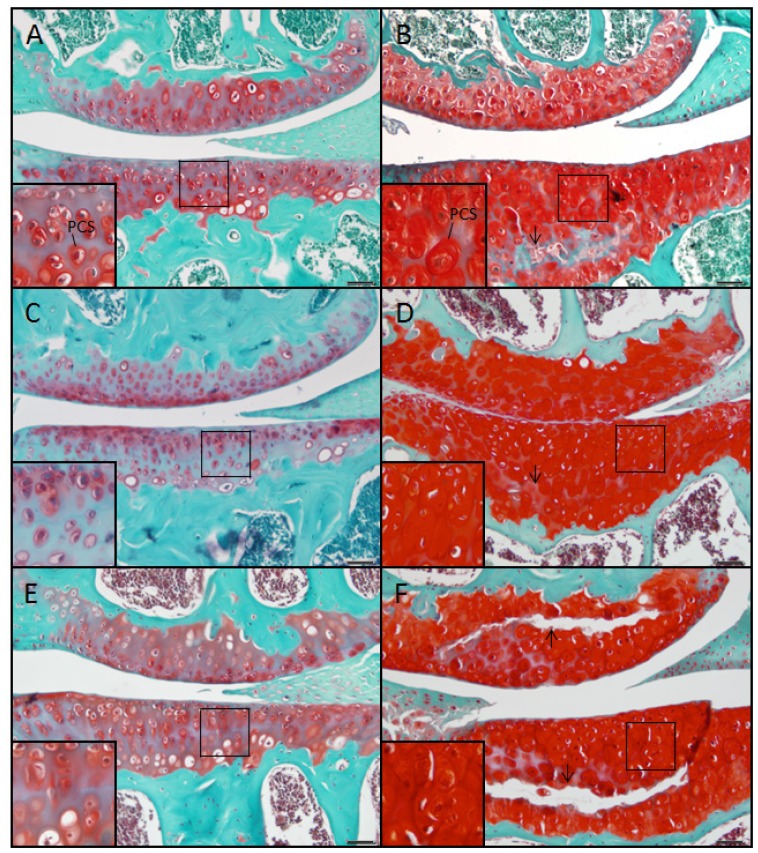
Staining with safranin O demonstrated a marked increase of proteoglycan within the pericellular space (PCS) of *sedc/sedc* (**B**,**D**,**F**) compared with age-matched +/+ murine knee joints (**A**,**C**,**E**) at 2 (**A**,**B**), 6 (**C**,**D**), and 9 (**E**,**F**) months of age. Note the horizontal fissuring in the region of the tidemark (arrows). Large panel images were taken at 200× magnification; insets at 400× (Bar = 50 μm).

At 2, 6 and 9 months the control mouse showed a smooth articular surface with no fibrillation. Due to the presence of proteoglycan, the chondrons stained moderately red with safranin O while the surrounding ECM stained reddish blue due to the dual presence of safranin O-positive proteoglycan and fast green-stained collagen (Figure 3A, inset). The chondrons were arranged into columns showing only moderate cell clustering.

#### Mutant (sedc/sedc)

In the mutant at 2, 6 and 9 months the chondrons stained intensely red with safranin O, and the PCS appeared swollen (Figure 3B, inset). The extent of fibrillation at the articular surface did not exceed that observed in age-matched controls. The ECM was diminished compared with control, but stained a reddish-blue as in the age-matched control. Chondrocyte arrangement was disordered and clustered, and cells were typically absent in the vicinity of the tidemark. As shown in H & E stained tissues, horizontal separation (fissuring) was observed at 9 months in the tidemark region, which in most cases extended vertically to the articular cartilage surface (Figure 3F).

### 2.3. The Homozygous Mutant Shows Early Onset and Increasing Severity of OA in the Knee Joint

**Figure 4:**
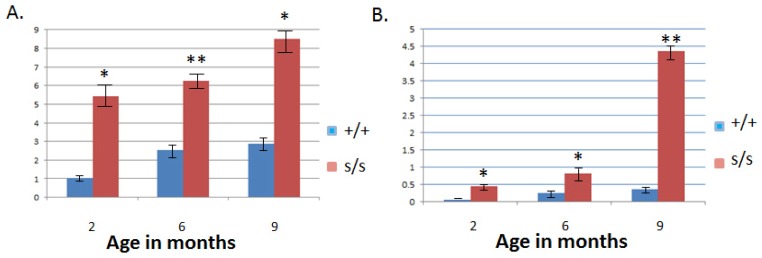
Scoring for joint pathology consistent with OA-like changes were performed using both the Modified Mankin and OARSI scoring systems. Mankin (**A**) and OARSI (**B**) scoring demonstrated a significant increase in the severity of OA-like changes in *sedc/sedc* compared with +/+ knee joints at 2, 6, and 9 month. (* *p* < 0.05; ** *p* < 0.01).

Both the modified Mankin [38] and Osteoarthritis Society International (OARSI) scoring [39] systems were used to quantify knee pathology. In both scoring systems histological sections from *sedc/sedc* mutants in comparison to wild type showed a significant increase in OA-like degradation as early as two months (*p* < 0.05). This distinction was observed through six and nine month mutant samples compared with their respective control counterparts (Figure 4). The progression of OA-like changes occurred more dramatically over time in the mutant compared to control. Both analytical systems were applied in this model to more accurately characterize joint pathology. The Mankin system was developed to examine severe OA originally. A modified version of Mankin scoring is widely used but might be less effective in assessing joints with minimal pathology. The OARSI system takes into account more parameters and divides the knee joint into quadrants for purposes of scoring. It is not surprising that significant OA-like changes were observed in both scoring systems in the unique *sedc/sedc* model.

### 2.4. Type II Collagen Is Present in *sedc/sedc* Articular Cartilage in both ECM and PCS

#### Wildtype control (+/+)

**Figure 5:**
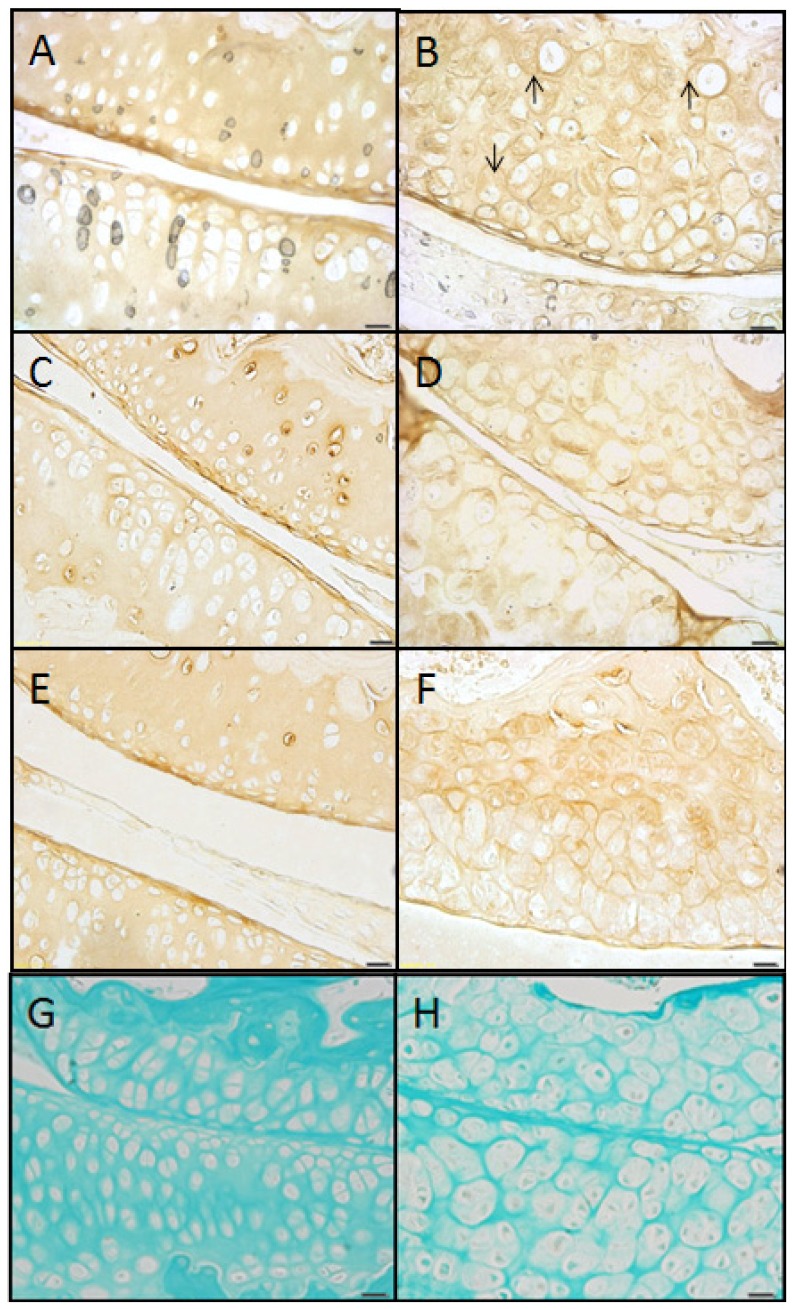
Staining with 1C10 antibody detected the presence of type II collagen in *sedc/sedc* (**B**,**D**,**F**) as was evident in age-matched +/+ murine knee joints (**A**,**C**,**E**) at 2 (**A**,**B**), 6 (**C**,**D**), and 9 (**E**,**F**) months of age. Two notable differences were observed between control and mutant tissue samples. First, we observed a greatly diminished territorial region of the ECM in the mutant compared to control at all ages. Second we note the presence of type II collagen in the expanded pericellular space in the mutant (arrows in **B**). Collagen presence was confirmed by staining with fast green (**G**,**H**). Images were captured at 200× magnification (Bar = 50 μm).

At all ages the articular cartilage of the control mouse showed positive staining for the 1C10 antibody indicating the presence of type II collagen (Figure 5A,C,E). Except for a few chondrons, which showed a rim of intense staining, most of the antibody was localized within the territorial region of the ECM compartment, leaving most chondrons relatively free of stain. Antibody staining was more intense at the articular surface. The control pattern of histochemical staining with fast green confirmed the pattern of antibody staining with 1C10 (Figure 5G).

#### Mutant (sedc/sedc)

As observed in the control at all ages, the mutant mouse stained positively with the 1C10 antibody, indicating the presence of type II collagen in the territorial region of the ECM but we note that this region is markedly decreased compared to the control (Figure 5B,D,F). Not only was the staining localized in the decreased area of territorial region of the ECM, it was observed in the enlarged PCS of most of the chondrons (Figure 5B, arrows). Interestingly, the enlarged PCS largely comprises the interstitial space in the mutant. The combination of fibril network and proteoglycan in the control provides cartilage with its characteristic firm nature that would be diminished in the mutant. The staining intensity was increased at the articular surfaces of both femoral and tibial regions in the mutant, as in the control. The mutant’s pattern of histochemical staining of collagen with fast green was consistent with the pattern of antibody staining with 1C10 (Figure 5H).

### 2.5. Evidence Confirming the Presence of Proteoglycan in the Expanded PCS of *sedc/sedc* Articular Cartilage

#### Wildtype control (+/+)

**Figure 6:**
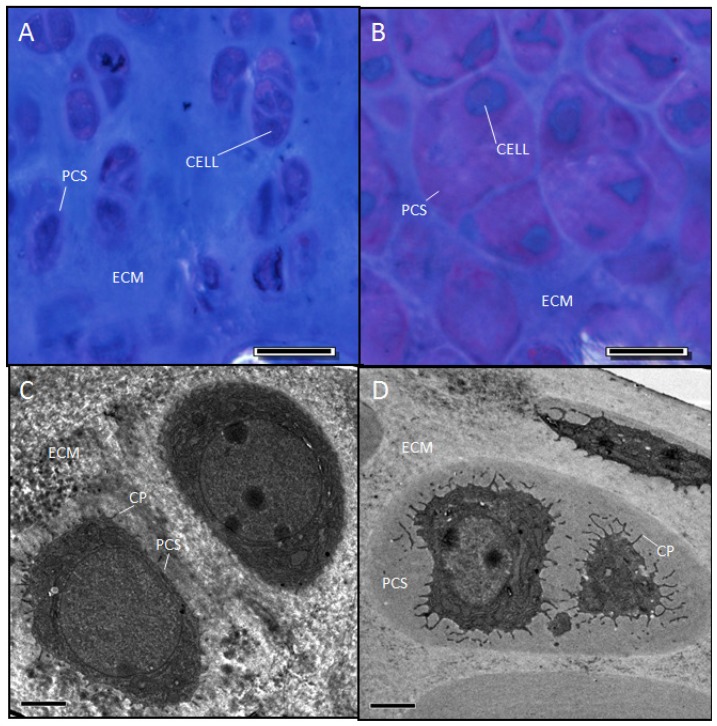
Two-month-old *sedc/sedc* mice show detectable metachromasia within the enlarged PCS (**B**) compared with +/+ mice (**A**). Electron microscopy revealed a non-fibrillar amorphous material within the enlarged PCS (D). Images were captured at 40× magnification (Bar = 20 μm) for light microscopy (**A**, **B**) and 2100× (Bar = 2 μm) for electron microscopy (**C**,**D**). Notably, *sedc/sedc* chrondrocytes show significantly elongated cytoplasmic processes (CP) compared with +/+ mice.

The ECM of resin-embedded articular cartilage for the control appeared as a homogenous light blue color following exposure to the toluidine blue/azure stain (Figure 6A), indicating the presence of proteoglycans. Correlating with the pattern of hematoxylin (see Figure 1A,C,E) and safranin O staining of control tissue (see Figure 3A,C,E), the presence of metachromasia within the PCS was only moderately detectable. Electron microscopy of control articular cartilage at low magnification revealed typical cells and only a small peripheral area of PCS (Figure 6C).

#### Mutant (sedc/sedc)

As in the control, the ECM of the mutant stained light blue (Figure 6B) indicating the presence of proteoglycan. Correlating with the staining pattern of hematoxylin (cf. Figure 1B,D,F) and safranin O (cf. Figure 3B,D,F), the expanded PCS of the mutant showed remarkable metachromatic staining at all ages indicating the strong presence of proteoglycan. Electron microscopy of mutant articular cartilage revealed cells bearing atypical cytoplasmic processes and surrounded by an expanded PCS containing amorphous material and lacking fibrils (Figure 6D). Even when fibrils were found, their diameter was not typical to controls. The decreased interstitial fibril meshwork coupled with enlarged PCS previously described would lead to abnormal cartilage. We presume that this critical combination of abnormalities would greatly affect the mechanical properties of the cartilage. Indeed, we are looking into the compression and shearing properties of mutant cartilage compared to control.

### 2.6. Collagen Fibril Diameter Is Smaller in *sedc/sedc* Articular Cartilage

#### Wildtype control (+/+)

**Figure 7:**
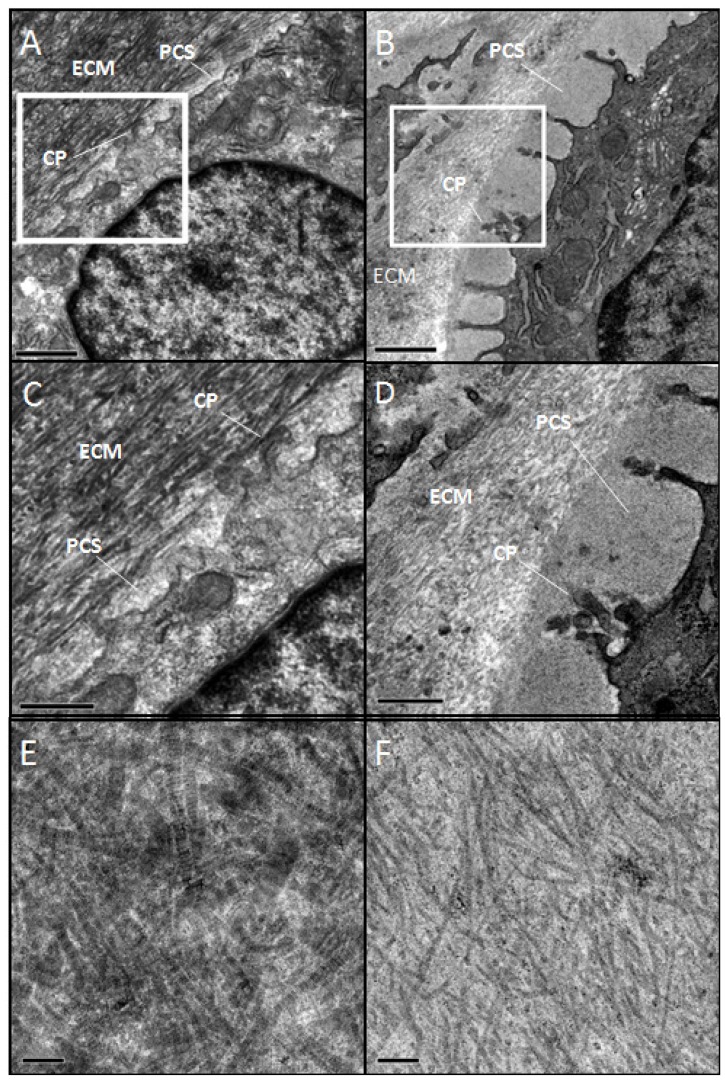
Electron microscopy revealed smaller diameter collagen fibrils in the extracellular matrix (ECM) of *sedc/sedc* compared with +/+ mice. At lower magnifications (2700× (**A**,**B**) and 6500× (**C**,**D**)), notable differences in *sedc/sedc* mice (**B**,**D**) are revealed in length of cytoplasmic processes (CP), pericellular space (PCS), and extracellular matrix (ECM) compared with +/+ mice (**A**,**C**). Images C and D show the area within the outlined box of images A and B. Micrographs of the ECM taken at 11,000x reveal the uniformly smaller diameter fibrils of the *sedc/sedc* (**F**) compared with +/+ mice (**E**). At lower magnification bars represent 1 μm, at higher magnification bars represent 0.5 μm. Micrographs of the 11,000 represent 0.1 μm.

**Figure 8:**
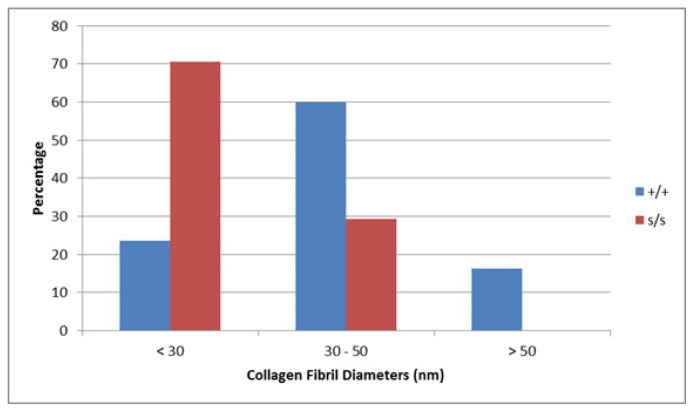
Measurement of collagen fibril diameter confirmed the smaller diameter fibrils in *sedc/sedc* mice shown in Figure 7. In the wild type the greatest abundance of fibrils measured between 30 and 50 nm, with some fibrils smaller than 30 and some greater than 50 nm, whereas in the mutant none were greater than 50 nm, some are in the 30 to 50 nm range but the highest percentage of fibrils was less than 30 nm diameter.

Wild type chondrocytes were surrounded by a limited PCS and showed small cellular protrusions or cytoplasmic processes (CP) (Figure 7A,C). Formed fibrils were evident in the ECM located outside the PCS (Figure 7A,C,E). These observations were consistent among cells in the various regions of the articular cartilage. The most abundant fibril diameter distribution was between 30 and 50 nm with some < 30 and some >50 nm (Figure 8).

#### Mutant (sedc/sedc)

Surrounding the chondrocyte, the mutant showed an enlarged PCS containing amorphous material. The chondrocytes themselves did not appear to be any larger in size than the control. The CPs extending toward the margin of the PCS were significantly longer in comparison to control (cf. Figure 7B,D *vs.* 7A,C). These findings were also observed in superficial as well as deep cells of mutant articular cartilage. At higher magnification, the mutant ECM displayed collagen fibrils that appeared much smaller in diameter than those of control (cf. Figure 7F*vs.* 7E). The majority of these fibrils (70.6%) averaged less than 30 nm, with some measuring between 30 and 50 nm (29.3%) and none measuring greater than 50 nm (Figure 8).

As described above we have demonstrated that the *sedc/sedc* mutant displays irregularities in the composition of ECM and PCS. We propose that such irregularities accelerate articular cartilage degradation and lead to early onset, non-load bearing, OA.

The increased proteoglycan staining in *sedc/sedc* mutants at all ages was primarily localized to the PCS. As previously mentioned, proteoglycans attract sodium and water due to their negative charge. This aids in giving the ECM its absorbent properties [25]. However, if these proteoglycans are unable to leave the PCS upon secretion from the chondrocyte, perhaps due to absence of a normal collagenous fibril structure, they cause the PCS to swell instead. We hypothesize that transport of proteoglycans from the PCS is coupled with excretion of type II collagen fibrils. Therefore, if collagen fibril formation is compromised, proteoglycan excretion from the PCS will likewise be disrupted. This explains the swollen, proteoglycan-rich PCS found in the *sedc/sedc* mutant, but absent in the control.

Type II collagen was found to be present in the *sedc/sedc* mutant as seen in the age-matched wild-type. However, the precursors of the collagen fibrils, collagen profibrils, are apparently ineffective at forming normal collagen fibrils as evidenced by the defective fibril diameter. This defect in fibril assembly would cause instability in the ECM that might lead to osmotic swelling of the proteoglycan-rich PCS. We predict that such swelling could compromise the ability of the chondrocyte to detect normal mechanical forces. This might explain why we observed no upregulation of the HtrA1, Ddr2, Mmp13 degradative pathway (unpublished observation) that has become a hallmark of OA progression [30,40]. It is possible that the *sedc/sedc* chondrocyte Ddr2 cell surface receptors might be unable to come in contact with mutant type II collagen fibrils because of the swollen PCS, thereby slowing down the typical process of destruction that takes place in the heterozygote *(sedc/+).*

## 3. Experimental Section

### 3.1. Experimental Animals and Determination of Genotype

Both +/+ control and *sedc/sedc* mutant mice were the products of crossed *sedc*/+ mice (JAX^®^ Mice # 003916 of The Jackson Laboratory, Bar Harbor, ME, USA). Animals were identified using an ear punch system. Animal maintenance and genotyping were performed under an approved IACUC protocol as previously described [30]. Essentially, genotyping was performed utilizing DNA isolated from tail biopsies followed by amplification via polymerase chain reaction, digestion with BtsCI to cleave the mutated nucleotide sequence, and visualization via agarose gel electrophoresis.

### 3.2. Tissue Processing

Tissues were processed as previously described [30]. Mice were euthanized at 2, 6, and 9 months of age by CO_2_ inhalation. Both right and left knees were excised and fixed in 4% paraformaldehyde followed by exposure to a formic acid solution for 2–3 weeks. The tissues were embedded in paraffin blocks with the anterior tibial surface flush with the cutting surface, and sectioned at 6 μm through the entire joint from the anterior to posterior surface.

### 3.3. Histological Evaluation of Articular Cartilage

Structural changes in articular cartilage were documented in tissues stained with hematoxylin and eosin (H & E), with sample sizes for +/+ and *sedc/sedc* knee joints at 2, 6 and 9 months equaling *n* = 3, 3; 2, 2 and 4, 1 respectively. Photographs were taken with a light microscope equipped with a digital camera. Adobe Photoshop CS was used to measure average cartilage thickness according to a predetermined area. These measurements allowed for a comparison of cartilage thickness between +/+ and *sedc/sedc* at different ages.

Analysis of tissues stained with safranin O and fast green for +/+ and *sedc/sedc* knee joints at 2, 6 and 9 months included sample sizes of *n* = 6, 7; 6, 6 and 9, 3 respectively. Articular cartilage from two representative sections of each stained slide was analyzed using both a modified Mankin and OARSI scoring system to quantify OA severity of each joint. Overall OARSI scoring was based on an osteoarthritic damage 0–6 subjective scoring system applied to all four quadrants of the knee as previously described [39].

As previously described [38] the Modified Mankin score is comprised of four categories: cartilage erosion score, chondrocyte periphery staining, spatial arrangement of chondrocytes, and background staining intensity. The overall Mankin score was applied to the knee joint as a whole and not to individual quadrants.

### 3.4. Collagen Described by Immunohistochemistry

Knee joints were harvested from +/+ and *sedc/sedc* mice at 2, 6 and 9 months and included sample sizes of *n* = 3, 3; 3, 2 and 2, 1 respectively. These samples were immediately embedded in Tissue-Tek^®^ OCT™ Compound and frozen in liquid nitrogen. The samples were stored at −80 °C until sectioning was performed. A cryostat was used to cut sections 12 μm thick at −20 °C. Immunohistochemistry was performed on sections for every sixth slide. The slides were stained using the collagen II monoclonal 1C10 primary antibody obtained from Drs. Russell Fernandes and David Eyre [41], followed by a secondary antibody of streptavidin/biotin. Diaminobenzidine (DAB) was utilized as the color reagent to visualize the presence of collagen proteins according to procedures previously reported except that we used hyaluronidase (Sigma H4272, St. Louis, MO, USA) [42]. Fast green staining of paraffin sections was used to confirm the presence of collagen in the ECM.

### 3.5. Localization of Proteoglycan by Light and Electron Microscopy

Following euthanasia, left knee joints were fixed in 2% glutaraldehyde-Na-cacodylate solution. The tissues were placed in a decalcification solution up to 7 days and then stained with osmium tetroxide and uranyl acetate to visualize lipids and nucleic acids, respectively. The samples were dehydrated in acetone and embedded in Spurr’s resin. Sections were cut 1μm thick and stained with a 1% toluidine blue-azure II solution for the purpose of tissue orientation and localization of proteoglycan as indicated by the presence of metachromasia. Resin-embedded samples were sectioned at 100 nm with a diamond knife and placed on a formvar/carbon-coated grid, then stained with lead citrate for visualization of cells and ECM under the electron microscope.

### 3.6. Determination of Collagen Fibril Diameter

From the above resin-embedded thin sections, chondrocytes and ECM were located and photographed using the electron microscope. Special attention was given to the structure of the pericellular space (PCS)—the region immediately surrounding the chondrocytes. Photographic images of collagen fibrils within the interterritorial space of the ECM were taken to determine fibril diameter.

### 3.7. Statistical Analysis

For cartilage thickness (superficial to deep) analysis, measurements were grouped according to +/+ or *sedc/sedc* mice at 2, 6, and 9 months of age. A two-tailed t test was performed comparing the control and experimental data within each age group. Results were considered statistically different at *p* < 0.05 significance.

A mixed models analysis of variance (ANOVA) was performed on the Modified Mankin and OARSI scored data. The dependent variables were the scores and the independent variables were age and genotype along with their interaction. Blocking was performed on each animal to account for their multiple measures. Post-hoc *t*-tests were performed to determine differences in genotype at each age. *p*-values equaling 0.05 or less were considered statistically significant.

Collagen fibril diameters were measured using ImageJ software. Images were taken of right knee articular cartilage from two mice of each genotype, and 30–50 collagen fibrils were counted per mouse. Measurements were statistically analyzed via the Least Squares Means test.

## 4. Conclusions

We conclude that the *sedc*/*sedc* is a unique model that can fill an important niche. There is currently a need for more extensive research regarding juvenile OA. Because the *sedc/sedc* mutant is dwarfed, early-onset OA, and non-load bearing, it provides a useful model for juvenile cartilage deficiencies. Therefore, we propose that it is an excellent model for Stickler’s syndrome in humans, especially in children and young adults. Since the mutant also exhibits unique matrix properties (*i.e.*, unique interstitial space and abnormal fibril makeup), it is an important model for both basic and applied studies on matrix biology.
